# Veterans Affairs Clinical Resource Hubs and Rates of Mental Health Community Care Referrals

**DOI:** 10.1001/jamanetworkopen.2025.60084

**Published:** 2026-02-27

**Authors:** Samantha L. Connolly, Erin L. Jaske, Chelle Wheat, Lawrence J. Wahlberg, Karin Nelson, Idamay Curtis, Bradford Felker

**Affiliations:** 1 Center for Health Optimization and Implementation Research, VA Boston Healthcare System, Massachusetts; 2 Department of Psychiatry, Harvard Medical School, Boston, Massachusetts; 3 Primary Care Analytics Team, VA Puget Sound Health Care System, Seattle, Washington; 4 Center for Veteran-Centered and Value-Driven Care, VA Puget Sound Health Care System, Seattle, Washington; 5 National Clinical Resource Hub, VA Central Office, Department of Veterans Affairs, Washington, DC; 6 Department of Psychiatry, School of Medicine, University of Colorado Denver, Aurora; 7 Department of Medicine, Division of General Internal Medicine, University of Washington School of Medicine, Seattle; 8 Department of Health Systems and Population Health, University of Washington School of Public Health, Seattle; 9 Department of Psychiatry and Behavioral Sciences, University of Washington School of Medicine, Seattle

## Abstract

**Importance:**

In an effort to increase access to care, the US Department of Veterans Affairs (VA) has created 2 additional pathways for veterans to receive mental health (MH) services: regional VA Clinical Resource Hubs (CRHs) and VA-purchased community care (CC). Previous studies have found that veterans rate their satisfaction with VA MH care, including via CRHs, higher than CC. It is unclear whether increased CRH use decreases referrals to CC.

**Objective:**

To determine whether clinics with higher CRH utilization have fewer CC referrals or, alternatively, whether clinics with local care shortages rely on both CRH and CC at similar levels to ensure adequate access to MH care.

**Design, Setting, and Participants:**

This longitudinal cohort study included all veterans with at least 1 outpatient MH encounter between 2018 and 2019 in 1149 MH clinics within the VA. The study evaluated changes in CC referral rates at clinics with and without CRH visits between baseline and post-CRH implementation (October 1, 2017, through September 30, 2023). In addition, CC referral rates at clinics with higher use of CRH were compared with clinics with lower CRH utilization. Data were analyzed from August 20, 2024, to July 25, 2025.

**Exposures:**

Clinics were classified based on whether they had any CRH utilization, as well as whether their per-patient rate of CRH visits fell in the top 25% (high penetration) or bottom 25% (low penetration).

**Main Outcomes and Measures:**

The main outcome was the number of CC referrals per 1000 patients. Difference-in-differences (DID) analyses were used.

**Results:**

The sample included 1 120 250 patients (mean [SD] age, 60.04 [15.38] years; 15.99% female). Clinics with any CRH utilization showed a slight but statistically significant increase in CC referrals in the post-CRH implementation period compared with clinics with no CRH utilization (DID, 0.525; 95% CI, 0.181-0.868; *P* = .003). However, clinics with high penetration of CRH had fewer CC referrals per month compared with low-penetration clinics at the end of the CRH implementation period (DID, –20.00 referrals; 95% CI, –21.90 to –18.20; *P* < .001).

**Conclusions and Relevance:**

In this cohort study of VA MH clinics, successful CRH implementation was associated with fewer CC referrals, suggesting that clinics with higher CRH utilization were less reliant on VA-purchased CC. Results underscore the importance of developing strong CRH infrastructures to ensure veteran access to high-quality MH care.

## Key Points

**Question:**

Is increased use of Clinical Resource Hubs (CRHs) associated with decreased referrals to community care (CC) for mental health care within the US Department of Veterans Affairs (VA)?

**Findings:**

In this cohort study of 1149 mental health clinics within the VA, clinics with greater use of CRHs had a mean of 20 fewer referrals to CC per 1000 patients per month.

**Meaning:**

Results of this study suggest that successful CRH implementation is associated with fewer CC referrals and that clinics with higher CRH utilization become less reliant on VA-purchased CC.

## Teaser

This cohort study reports on the association of veterans’ use of Department of Veterans Affairs’ Clinical Resource Hubs for mental health care with the number of referrals to community care.

## Introduction

Ensuring that veterans have access to high-quality mental health (MH) care is a key priority of the US Department of Veterans Affairs (VA), the nation’s largest integrated health care system. In addition to providing MH services to veterans at nearby VA facilities, VA has expanded access by offering 2 additional care pathways: VA Clinical Resource Hubs (CRHs) and VA-purchased community care (CC). CRHs are regionally based telehealth contingency staffing hubs through which MH clinicians deliver virtual care to veterans at facilities experiencing MH staffing shortages. CRHs were developed in response to a congressional mandate for VA to increase access to care via the VA Maintaining Internal Systems and Strengthening Integrated Outside Networks (MISSION) Act. The implementation of these hubs began in 2019, and 1 hub now exists in each of the 18 VA geographic regions.^1^ The VA also increased access to care via its VA-purchased CC program, in which the VA pays for veterans to receive MH services from a non-VA clinician in their local community. Access to CC increased when the Veterans Choice Act was passed by Congress in 2014, and eligibility expanded further when the MISSION Act was signed into law in 2018.^2^ The VA can pay for CC if any of the following conditions are met: (1) the veteran needs a service that is not provided at a VA facility, (2) the veteran lives in a state or territory that does not have a full-service VA health facility, (3) it is in the veteran’s best medical interest, (4) VA cannot provide that service in a way that meets its quality standards, or (5) VA cannot provide care within its standards for drive and wait times (<30-minute average drive time or <20-day wait time for an MH appointment).^3^

Findings comparing the relative quality of VA MH services and CC have been mixed. In 1 study^4^ comparing patients who received care via CRHs with those who received CC, those in the CRH group reported substantially fewer barriers to access, while satisfaction scores and clinical outcomes did not differ between the groups. However, a recent survey^5^ found that veterans rated the overall quality of their MH clinician as lower when receiving CC compared with either CRH or non-CRH VA care, and another study^6^ found that CC behavioral health clinicians had fewer years of training compared with VA clinicians. An additional study^7^ found that veterans with MH conditions had significantly lower satisfaction ratings for CC compared with those without MH conditions, although the study did not compare ratings with VA care. There may be challenges coordinating care between VA and CC, which could impact quality.^2^ In addition, compared with VA clinicians, CC clinicians may be less likely to have received training in topics such as military culture and MH conditions that are more prevalent among veterans, including posttraumatic stress disorder, which may impact quality of care.^2^ Qualitative analyses found that veterans tended to prefer receiving their care within the VA when possible, although some respondents emphasized the importance of being able to choose where they received their care.^8^

Given these potential differences in quality and preference, it is important to examine whether the relative use of CRH is associated with CC MH services within clinics. Both programs are intended to increase the availability of MH care, particularly at sites that may be experiencing staffing shortages or longer wait times. It is possible that clinics with higher CRH utilization have fewer CC referrals, as these clinics may have developed stronger infrastructures for CRH referrals and are able to accommodate more of their patients via this pathway, thereby decreasing the need for CC. Alternatively, clinics with local care shortages may rely on both CRH and CC at similar levels to ensure adequate access to services for their patients. This study assesses whether use of VA CRH for MH is associated with number of referrals to CC.

## Methods

This retrospective longitudinal cohort study analyzed MH CC referrals from October 1, 2017, through September 30, 2023, across all VA clinics. Data were analyzed from August 20, 2024, to July 15, 2025. This evaluation is part of an ongoing quality-improvement effort at the VA and is not considered research activity as determined by the Office of Primary Care; thus, it is not subject to institutional review board review and waivers and informed consent were not required. This study follows the Strengthening the Reporting of Observational Studies in Epidemiology (STROBE) reporting guideline for observational studies and includes all required information as outlined in the STROBE Cohort Study checklist.^9^

The cohort included 1149 VA clinics and 1 120 250 enrolled veterans with at least 1 VA outpatient MH encounter during the pre-CRH implementation period (2018-2019). CC referrals, patient demographics (including age, gender, self-reported race and ethnicity^10^), marital status, priority status,^11^ rurality, drive distance to the nearest primary care clinic, Gagne score,^12^ and MH comorbid conditions), and clinic-level covariates (including clinic size, primary care staffing ratio,^13^ flags for community-based outreach clinics, MH underserved scores,^14^ medically underserved scores, and measures of MH population covered [eTable 1 in Supplement 1] and MH staffing [eTables 2 and 3 in Supplement 1]) were obtained via the VA Corporate Data Warehouse. Race and ethnicity were included because racially and ethnically minoritized people have been shown to have lower rates of mental health utilization and access.^15^ Clinics were considered engaged in CRH if they had 5 or more CRH MH encounters for at least 2 consecutive months. Each patient was assigned an indicator if they received care at a site offering CRH MH care. Data were aggregated at the facility level, and clinics were classified as either using CRH or being a non-CRH site. A measure of the percentage of clinic-level MH care that was delivered by CRH (the CRH MH–penetration score) was used to assess the amount of CRH MH care that clinics were receiving relative to other CRH clinics. CRH-engaged clinics were assigned an indicator if their CRH MH–penetration scores were among the top 25% (high CRH penetration) or bottom 25% (low CRH penetration). This method has been used in previously published CRH analyses.^10^

### Statistical Analysis

Descriptive comparisons between CRH and non-CRH clinics, and between high and low CRH MH–utilizing clinics, were conducted using *t* tests and χ^2^ tests. Clinics were weighted and matched using inverse probability weighting, adjusting for key covariates that may differ between groups in an effort to prevent confounding, including proportion of the MH population covered (eTable 1 in Supplement 1), MH staffing levels (eTables 2 and 3 in Supplement 1), proportion of rural and female veterans, facility type (VA medical center vs community-based outpatient clinic), region, the number of monthly CC referrals at baseline, clinic size, and the number of MH visits at baseline. Covariates were chosen given that they have shown significant differences based on CRH MH site status in previous studies.^10^ The estimates from a logistic regression model were used to generate propensity scores and subsequent inverse probability weights. Covariate balance was assessed before and after weighting using the cobalt package in RStudio 2025.05.1 Build 513(R Foundation for Statistical Computing) with good overall balance (standardized mean difference < 0.10). No weight stabilization or truncation was needed.

We applied a difference-in-differences (DID) approach to evaluate changes in CC MH referral rates for CRH MH–engaged clinics vs non–CRH MH-engaged clinics between the pre–CRH implementation period (2018-2019) and post–CRH MH implementation (2020-2023) using the propensity-matched cohorts. Specifically, we used the Puhani DID estimator to estimate the pre-post change in outcome and calculated SEs using the delta method.^16^ We assessed the parallel trends assumption by visual inspection of graphical trends of the outcome, as well as by estimating regressions that included a linear time trend, group assignment, and the interaction between time trend and group assignment. Although there is no direct way to test if the parallel trends assumption holds, visual inspection of trend graphs and no evidence of an interaction between group assignment and time in the logistic regression model provide evidence in support of this assumption. We then used the same approach to evaluate changes in CC MH referral rates for high CRH MH–penetration vs low CRH MH–penetration sites. Two-sided *P* values < .05 were considered statistically significant. Analyses were conducted using SAS EG version 9.3 (SAS Institute) and RStudio 2025.05.1 Build 513 (R Foundation for Statistical Computing).

## Results

The overall study sample included 1 120 250 patients (mean [SD] age, 60.04 [15.38] years; 15.99% female and 84.01% male). Self-reported race and ethnicity categories were American Indian or Alaskan Native, 1.06%; Asian, 1.27%; Hispanic, 10.11%; Native Hawaiian, 0.77%; Non-Hispanic Black, 25.83%; Non-Hispanic White, 57.66%; Other Pacific Islander, 0.10%; and multiple races or other, 2.13%.

### CRH vs Non-CRH Clinics

**Figure 1.**
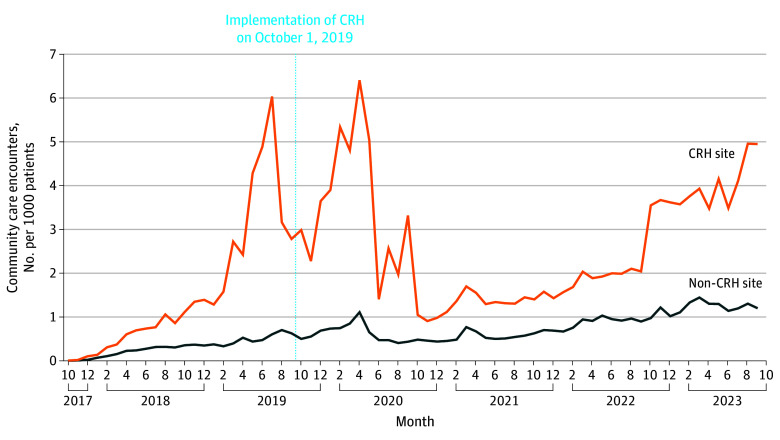
Line Graph of Monthly Trends of Community Care MH Referrals Before and After CRH MH Implementation at Non–CRH-Engaged vs CRH-Engaged Clinics CRH indicates Clinical Resource Hub; MH, mental health.

At baseline, clinics that used CRH MH services (n = 419) were larger (mean [SD] clinic size, 8192.79 [8059.80] vs 4553.69 [4617.31] unique enrolled patients; *P* < .001), had fewer mean (SD) MH visits per patient (2.978 [1.869] vs 2.517 [2.364]; *P* < .001), and were more likely to be medical centers vs community-based outpatient clinics (58% community-based clinics vs 68% medical centers; *P* < .001) compared with clinics that were not engaged with CRH MH (n = 730). Patients at CRH MH–engaged clinics had significantly higher mean (SD) rates of several comorbid MH conditions, including bipolar disorder (9.47% [4.12%] vs 7.91% [4.95%]; *P* < .001), depression (48.46% [8.31%] vs 46.62% [11.55%]; *P* < .001), and substance use disorder (25.05% [10.97%] vs 21.02% [12.54%]; *P* < .001) compared with clinics that were not providing CRH care (Table 1). At baseline, CRH-engaged clinics referred more patients to CC compared with non-CRH clinics (mean [SD] 6.38 [22.39] referrals/1000 patients at CRH clinics vs 3.08 [10.84] referrals/1000 patients at non-CRH clinics; *P* < .001). We then used DID analysis to compare preintervention and postintervention referral rates between CRH MH clinics and non-CRH MH clinics to see how the number of referrals changed following CRH implementation efforts. CRH MH clinics generated a mean (SD) of 0.53 (0.43) more CC MH referrals in the postimplementation period (DID, 0.525; 95% CI, 0.181-0.868; *P* = .003) compared with non–CRH MH clinics (Figure 1, Table 2).

**Table 1.** Baseline Differences Between Non–CRH-Engaged Clinics and CRH-Engaged Clinics^a^

Characteristic	Non–CRH-engaged clinic (n = 730)	CRH-engaged clinic (n = 419)	*P* value^b^
Patient characteristics (n = 1 120 250)			
Age, y	57.40 (4.25)	56.72 (3.14)	.001
Sex, %			
Female	14.76 (6.38)	14.75 (4.46)	.20
Male	78.08 (8.64)	78.11 (6.21)	.70
Race and ethnicity, %^c^			
American Indian or Alaska Native	2.17 (9.38)	1.70 (4.25)	.33
Asian	0.89 (2.07)	1.00 (2.12)	.76
Hispanic	8.59 (13.11)	8.13 (10.82)	.55
Native Hawaiian	0.52 (0.62)	1.38 (6.99)	.001
Non-Hispanic Black	18.36 (19.55)	15.14 (17.45)	.01
Non-Hispanic White	66.42 (23.36)	69.25 (21.72)	.04
Other Pacific Islander	0.09 (0.21)	0.10 (0.16)	.76
Multiple races/other^d^	1.98 (1.63)	2.29 (1.59)	.002
Marital status, %			
Married	46.13 (13.66)	43.77 (10.50)	<.001
Not married	52.67 (13.83)	54.77 (10.61)	<.001
Priority status, %			
No disability	5.37 (3.22)	5.52 (2.39)	.005
Low income	16.08 (11.15)	17.83 (7.85)	<.001
Low/moderate disability	17.46 (6.55)	18.51 (5.48)	<.001
High disability	53.82 (13.38)	50.48 (10.98)	<.001
Socioeconomic status decile	4.13 (1.26)	4.15 (0.99)	>.90
Rurality, %			
Urban	51.05 (31.42)	49.66 (28.45)	.20
Rural	34.89 (27.39)	36.44 (24.79)	.069
Highly rural/insular islands	6.34 (13.12)	6.49 (11.57)	<.001
Drive distance to PC clinic, miles	17.64 (18.37)	17.54 (15.93)	.20
Gagne score	0.53 (0.43)	0.58 (0.26)	<.001
History of homelessness, %	5.10 (8.30)	6.39 (6.59)	<.001
Comorbid conditions, %			
ADHD	3.4 (2.2)	4.1 (2.2)	<.001
Anxiety	33.33 (10.15)	34.31 (7.33)	.035
Bipolar disorder	7.91 (4.95)	9.47 (4.12)	<.001
Cognitive disorder	6.40 (5.13)	6.75 (2.94)	<.001
Depression	46.62 (11.55)	48.46 (8.31)	.009
PTSD	38.12 (13.58)	39.54 (9.12)	.030
Substance use disorder	21.02 (12.54)	25.05 (10.97)	<.001
Schizophrenia	3.22 (5.84)	3.37 (3.07)	<.001
Traumatic brain injury	1.19 (18.50)	1.27 (1.05)	.002
Utilization			
MH visits	2.517 (2.364)	2.978 (1.869)	<.001
PCMHI visits	0.51 (1.00)	0.48 (0.78)	.010
Primary care visits	1.30 (0.40)	1.33 (0.37)	.20
PCMM visits	10.61 (4.24)	11.03 (2.57)	<.001
Outpatient visits	10.75 (4.25)	11.20 (2.58)	<.001
Clinic characteristics			
Clinic size, No. of enrolled unique patients	4553.69 (4617.31)	8192.79 (8059.80)	<.001
PC staffing ratio	3.06 (1.09)	3.05 (1.07)	.60
CBOC, No./total No. (%)	499/730 (68)	245/419 (58)	<.001
MH underserved score, No./total No. (%)	71/724 (9.8)	96/418 (23)	<.001
Medically underserved areas	0.07 (0.09)	0.08 (0.10)	<.001
MH population coverage^e^	0.02 (0.44)	−0.12 (0.45)	<.001
Outpatient MH staff to patient ratio^e^	7.03 (1.30)	7.33 (1.46)	.002
Outpatient MH population staffing ratio^e^	1.96 (0.43)	1.94 (0.46)	.061
Community care referrals count/1000 unique patients	3.08 (10.84)	6.38 (22.39)	<.001

Abbreviations: ADHD, attention-deficit/hyperactivity disorder; CBOC, community-based outpatient clinic; CRH, Clinical Resource Hub; MH, mental health; PC, primary care; PCMHI, primary care mental health integration; PCMM, primary care management module; PTSD, posttraumatic stress disorder.

^a^ Values are reported as mean (SD) unless otherwise indicated.

^b^ Derived by using Wilcoxon rank sum test or Fisher exact test.

^c^ Race and ethnicity were self-reported.

^d^ Includes multiple races and any time people self-reported as other from Veterans Affairs administrative data.

^e^ eTable 1 in Supplement 1 gives details on population coverage; eTable 2 in Supplement 1 defines outpatient MH staff to treated patient ratio, and eTable 3 in Supplement 1 defines outpatient MH population staffing ratio.

**Table 2.** DID Analysis of the Number of Community Care Referrals Before and After CRH MH Implementation

Model	DID (95% CI)	SE	*P* value
CRH MH-engaged clinics vs non–CRH MH-engaged clinics	0.525 (0.181 to 0.868)	0.175	.003
High-penetration CRH MH clinics vs low-penetration CRH MH clinics	–20.00 (–21.90 to –18.20)	0.943	<.001

Abbreviations: CRH, Clinical Resource Hub; DID, difference in differences; MH, mental health.

### High vs Low CRH–Penetration Clinics

**Figure 2.**
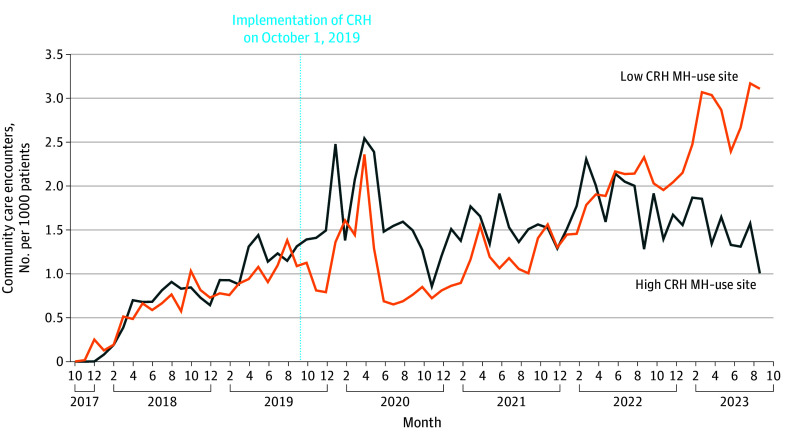
Line Graph of Monthly Trends of Community Care Mental Health (MH) Referrals Before and After Clinical Resource Hub (CRH) MH Introduction at Low CRH MH–Penetration vs High CRH MH–Penetration Clinics

At baseline, high-penetration CRH MH clinics (n = 115) had mean (SD) fewer MH visits (19.3 [9.0] vs 43.1 [20.9]; *P* < .001), were more likely to be rural (51.1% [25.7%] vs 23.3% [17.8%]; *P* < .001), and were more likely to be community-based outpatient clinics (70% vs 31%; *P* < .001) compared with the low-penetration CRH MH clinics (n = 121). Patients at low CRH MH–penetration clinics had higher, significant percentages of several MH comorbid conditions, including bipolar disorder (10.8% [3.2%] vs 8.2% [4.6%]; *P* < .001), substance use disorder (32.8% [11.7%] vs 20.5% [8.9%]; *P* < .001), and schizophrenia (5.1% [3.6%] vs 2.1% [2.8%]; *P* < .001) compared with high CRH MH–penetration clinics. Clinics with high CRH MH penetration had small but significant differences in CC referral rates at baseline compared with clinics with low CRH MH penetration (mean [SD], 4.51 [8.64] referrals/1000 patients at low-penetration CRH MH clinics vs 5.29 [6.28] referrals/1000 patients at high-penetration CRH MH clinics; *P* < .001; Table 3). DID models showed that clinics with high CRH MH penetration experienced a significant reduction in CC MH referrals in the postimplementation period, generating, on average, 20 fewer referrals per month compared with low-penetration clinics (DID, –20.00; 95% CI, –21.90 to –18.20; SE, 0.94; *z* score, –21.20; *P* < .001) (Table 3, Figure 2).

**Table 3.** Baseline Differences Between Low CRH MH–Penetration and High CRH MH–Penetration Clinics^a^

Variable	Low CRH MH–penetration clinic (n = 121)	High CRH MH–penetration clinic (n = 115)	*P* value^b^
Patient characteristics (n = 259 950)			
Age, y	56.33 (2.26)	57.27 (4.13)	.049
Sex, %			
Female	14.36 (3.46)	14.49 (5.60)	.90
Male	78.52 (4.66)	78.67 (7.88)	.50
Race and ethnicity,^c^ %			
American Indian or Alaska Native	1.17 (1.95)	3.34 (7.81)	.03
Asian	1.18 (1.68)	0.79 (2.43)	.12
Hispanic	9.11 (12.23)	8.07 (9.58)	.44
Native Hawaiian	0.61 (0.53)	2.78 (12.33)	.11
Non-Hispanic Black	21.59 (17.61)	8.97 (16.68)	<.001
Non-Hispanic White	63.07 (20.59)	73.76 (22.43)	<.001
Other Pacific Islander	0.11 (0.13)	0.13 (0.27)	.09
Multiple races/other^d^	2.08 (0.76)	2.80 (2.40)	.01
Marital status, %			
Married	36.55 (9.34)	50.21 (10.34)	<.001
Not married	62.12 (9.59)	48.13 (10.05)	<.001
Priority status, %			
No disability	4.88 (1.50)	6.39 (3.27)	<.001
Low income	19.69 (7.29)	17.61 (9.24)	.007
Low/moderate disability	17.10 (3.87)	21.31 (7.21)	<.001
High disability	50.74 (9.06)	47.33 (13.07)	.04
Rurality, %			
Urban	66.08 (21.56)	28.35 (26.30)	<.001
Rural	23.25 (17.78)	51.09 (25.69)	<.001
Highly rural/insular islands	3.45 (6.07)	12.95 (17.79)	<.001
Drive distance to PC clinic, miles	12.79 (4.66)	28.30 (27.08)	<.001
Gagne score	0.73 (0.24)	0.43 (0.25)	<.001
Socioeconomic status decile	4.14 (1.00)	4.05 (1.16)	.40
History of homelessness, %	11.29 (7.41)	3.24 (4.29)	<.001
Comorbid conditions, %			
ADHD	3.76 (1.87)	3.24 (2.12)	.002
Anxiety	34.79 (6.70)	32.65 (7.40)	.03
Bipolar disorder	10.75 (3.23)	8.16 (4.57)	<.001
Cognitive disorder	7.16 (2.46)	6.00 (3.19)	<.001
Depression	48.26 (6.82)	47.01 (8.70)	.60
PTSD	38.24 (7.30)	40.13 (10.15)	.30
Substance abuse disorder	32.84 (11.70)	20.52 (8.92)	<.001
Schizophrenia	5.08 (3.55)	2.07 (2.75)	<.001
Traumatic brain injury	1.39 (0.72)	1.20 (1.21)	<.001
Utilization			
MH visits	4.312 (2.085)	1.927 (0.897)	<.001
PCMHI visits	0.41 (0.24)	0.45 (0.60)	.005
Primary care visits	1.30 (0.24)	1.36 (0.49)	.60
PCMM visits	12.51 (2.55)	9.76 (2.25)	<.001
Outpatient visits	12.71 (2.58)	9.93 (2.19)	<.001
Clinic characteristics			
Clinic size, No. of enrolled unique patients	12 623.37 (8677.29)	2825.16 (3399.95)	<.001
PC staffing ratio	2.82 (0.66)	3.32 (1.76)	.04
CBOC, No./total No. (%)	29/95 (31)	74/105 (70)	<.001
MH underserved score, No./total No. (%)	14/94 (15)	41/105 (39)	<.001
Medically underserved areas	0.11 (0.08)	0.05 (0.10)	<.001
MH population coverage^e^	0.04 (0.51)	−0.24 (0.43)	<.001
Outpatient MH staff to patient ratio^e^	7.56 (1.58)	7.54 (1.62)	.80
Outpatient MH population staffing ratio^e^	2.10 (0.54)	1.89 (0.50)	.001
Community care referrals/1000 unique patients	4.51 (8.64)	5.29 (6.28)	.002

Abbreviations: ADHD, attention-deficit/hyperactivity disorder; CBOC, community-based outpatient clinic; CRH, Clinical Resource Hub; MH, mental health; PC, primary care; PCMHI, primary care mental health integration; PCMM, primary care management module; PTSD, posttraumatic stress disorder.

^a^ Values are reported as mean (SD) unless otherwise indicated.

^b^ Derived by using Wilcoxon rank sum test or Fisher exact test.

^c^ Race and ethnicity were self-reported.

^d^ Includes multiple races and any time people self-reported as other from Veterans Affairs administrative data.

^e^ eTable 1 in Supplement 1 gives details on population coverage; eTable 2 in Supplement 1 defines outpatient MH staff to treated patient ratio, and eTable 3 in Supplement 1 defines outpatient MH population staffing ratio.

## Discussion

The current study examined whether increased use of VA CRHs was associated with decreased CC referrals for veterans receiving MH services. When comparing clinics with any CRH MH referrals with clinics with no CRH MH referrals, there was a slight but significant increase in CC referrals among CRH MH–engaged clinics in the postimplementation period. These findings suggest that clinics falling into the *any* CRH MH utilization category are generally underresourced facilities that may turn to both CRH and CC for referral support. However, when classifying CRH clinics as either high or low penetration based on their relative utilization of CRH MH visits (top or bottom 25%), a substantially larger effect emerged in the opposite direction. Specifically, high-penetration CRH clinics had a mean (SE) of 20 (0.943) fewer CC encounters per month compared with low-penetration clinics at the end of the CRH implementation period. Clinics that utilize CRHs are, by definition, sites with MH access shortages. However, our findings demonstrate an important difference based on whether sites were classified based on having any CRH use vs having CRH use that fell in the top 25% (high-penetration sites). Use of the any CRH use definition identified underresourced sites more generally that rely on both CRH and CC, but this definition was not sensitive enough to determine whether CRH referrals had been fully integrated into site processes. Conversely, the high-penetration definition identified a unique subset of sites that made CRH referrals more frequently and demonstrated substantial decreases in CC referrals over time. This finding suggests that successful implementation of CRHs may be associated with decreased reliance on CC.

These results underscore the importance of having strong infrastructures and adequate staffing to support the development of CRHs and achieve optimal rates of uptake. The rollout of CRHs included a bundle of implementation strategies, such as mandating program elements, involving leadership, engaging in strategic planning, providing national trainings, and developing tools to collect feedback and track progress.^1,17^ While these strategies were offered to all clinics, the difference in CRH penetration rates observed in the current study suggests that there was variability in implementation success across facilities. Similarly, clinics may differ in the degree to which CC referral processes have been successfully implemented; indeed, some clinics may struggle with identifying adequate networks of CC clinicians and may encounter challenges and confusion around reimbursement and eligibility requirements, while other clinics may face fewer barriers and may therefore use CC more frequently.^18^ High CRH–penetration clinics were more likely to be smaller, community-based outpatient clinics in rural locations. These site-level characteristics may allow CRH MH services to more rapidly integrate into the routine workflow of these smaller clinics, although additional research is needed to better understand what factors led to these sites utilizing CRHs at greater rates relative to CC. Future, more in-depth work will be necessary to understand factors contributing to site-level differences in both CRH and CC uptake.

The findings have relevance for non-VA settings as well. Multiple other health systems, including those serving rural and low-income populations, have turned to telemental health hub models to meet patient demand; via these programs, patients receive telemental health care from an alternative facility within the same or affiliated health system.^19,20^ Current findings lend support to the premise that these models can be successful in retaining patients within health systems. This is particularly important as receiving MH services outside of one’s primary health network has been found to have higher costs and lead to more fragmented care.^21,22,23^ The development of robust telehealth hub models may serve to positively benefit MH care provision in a wide variety of settings, both within and outside of the VA.

### Strengths and Limitations

Strengths of this work include its use of rigorous statistical methods to examine differences between sites with high and low CRH utilization. We utilized inverse probability weighting, which accounted for covariates that may differ between groups, such as clinic size, staffing, and rurality, in an effort to reduce potential confounding.

The current study also has several limitations. First, as a retrospective observational study, it cannot establish causality and unmeasured confounding may affect findings. Clinics were labeled as CRH sites if they had 2 or more consecutive months of use within a fiscal year, which may not fully capture the consistency or integration of CRH MH services across clinics. CC referrals were used as a proxy for unmet demand, but this metric may reflect administrative factors or patient preference rather than actual MH access gaps. The current study did not differentiate between therapy and medication management visits. It will be important to examine whether there are differences between high and low CRH–utilizing sites with regard to the types of appointments most frequently offered. It is also worth noting that the postimplementation phase of the study occurred during the COVID-19 pandemic. However, as CRH implementation was a nationwide initiative, we would not expect that CRH clinics would be differentially impacted by COVID-19 compared with non-CRH clinics. Finally, while this study describes patterns of utilization, it did not evaluate clinical outcomes or patient satisfaction, which are critical for understanding the effectiveness of the CRH program. However, recent work has demonstrated higher patient ratings of care quality for CRH compared with CC,^5^ and other studies have shown advantages of VA MH care over CC, including greater patient satisfaction^7^ as well as the availability of more highly trained clinicians within VA.^6^ Qualitative work has also found that veterans tended to prefer receiving care within VA, although the ability to choose the location of care was also deemed to be important.^8^

## Conclusions

Findings of this study suggest that successful implementation of CRHs, VA’s regional telehealth contingency staffing program, was associated with decreased CC utilization. Given that veterans may rate their satisfaction with VA MH services, including those provided via CRHs, as higher than CC, these results emphasize the importance of supporting clinics in developing strong CRH infrastructures. As veterans now have an increasing degree of choice in how they receive their MH care, it will be critical to continue studying factors that impact the relative utilization of VA MH services and CC.

## Notes

Supplement 1. **eTable 1.** Mental health population coverage definition **eTable 2.** Outpatient MH staff to treated patient ratio definition **eTable 3.** Outpatient MH population staffing ratio definition

Supplement 2.
Data Sharing Statement
