# Supplementary material for: Veterans Affairs Clinical Resource Hubs and Rates of Mental Health Community Care Referrals
Source: JAMA Netw Open. 2026 Feb 27;9(2):e2560084. doi: 10.1001/jamanetworkopen.2025.60084 (PMC12949438; doi:10.1001/jamanetworkopen.2025.60084)
Supplement: Supplement 1. — eTable 1. Mental health population coverage definition eTable 2. Outpatient MH staff to treated patient ratio definition eTable 3. Outpatient MH population staffing ratio definition [file jamanetwopen-e2560084-s001.pdf]

Supplemental Online Content

Connolly SL, Jaske EL, Wheat C, et al. Veterans Affairs clinical resource hubs and rates of mental health community care referrals. *JAMA Netw Open*. 2026;9(2):e2560084
doi:10.1001/jamanetworkopen.2025.60084

eTable 1. Mental health population coverage definition

eTable 2. Outpatient MH staff to treated patient ratio definition

eTable 3. Outpatient MH population staffing ratio definition

This supplemental material has been provided by the authors to give readers additional information about their work.

eTable 1

Electronic Technical Manual (eTM) Measure Specification

MH Population Coverage (pcov2)

tablename	Attribute	Information
Definition	Measure Name	MH Population Coverage
	Measure Mnemonic	pcov2
	Measure Coordinator	Ann S. Combs
	Measure Long Description	Composite score based on 16 population-coverage measure z-scores (weight=1 except where noted): Fam2; HIAS21; HIAS72; PACT21 (.5); PCMHI7 (.5); Pmed1; Psy32; Psy34; Psy36; Psy38; PTSD56 (.25); PTSD57 (.75); SMIE1; SUD4; SUD16; PopAx
	Date of Activation	Nov 16 2015
	Date of Deactivation	
	Data Source Definition	
	Data Source Report	PEC Portal Tools
	Measure Notes	FY21Q4: PTSD56 and PTSD57 will be weighted at .25 and .75 respectively. In response to HEDIS updates, MPT1 and

		IAD1 retired and replaced by DMH1 and DSU1. DMH1 and DSU1 were folded into newly created PopAx1 along with Pop6 and PMH1 (previously available on other reports).<br><br>FY20Q4: The PTSD56 metric will be piloted with a new companion metric, PTSD57 which will also be reported with Population Coverage metrics, but not weighted (weight=0). PCMHI6 will be replaced by PCMHI7.<br><br>FY16Q4: Replaces pcov2 in the SAIL beginning in FY16Q4 to reflect revisions in constituent measures in response to field recommendations. SMIE2 has been dropped. HIAS21 and HIAS72 have been modified to credit high performing facilities that offer non-mandated PRRC or MHICM programs.
Details	Measure - Indicator Type	National Indicator
	Target Definition	
	Scoring and Report Display	Rolling 12 Month
	Preferred Score Direction	Higher is Better
	Score Calculation Definition	Facility scores on the 16 constituent measures are first standardized based on last fiscal year`s Q4 results. Standardized scores less than -3.00 are rounded to -3.00, and scores greater than 3.00 are rounded to 3.00. Standardized scores are then averaged with the following weighting: Psy32 (1); Psy34 (1); Psy36 (1); Psy38 (1); Pmed1 (1); PopAx (1); PACT21 (.5); PCMHI7 (.5); PTSD56 (.25); PTSD57 (.75); HIAS21 (1); HIAS72 (1); SMIE1 (1); SUD4 (1); SUD16 (1); Fam2 (1).
	FY Specific Annotations	FY21Q4: PTSD56 and PTSD57 are no longer pilots and weighted at .25 and .75 respectively. In response to HEDIS updates, MPT1 and IAD1 retired and replaced by DMH1 and DSU1. DMH1 and DSU1 were folded into newly created PopAx1 along with Pop6 and PMH1 (previously available on other reports). PTBISE1 is an unweighted pilot being assessed in partnership with PTBISE3 in Continuity of Care Composite.<br><br>PY21 Updates: PTSD56 and new companion measure, PTSD57, are being pilot tested and will not be weighted in PCOV2 calculations this PY. PCMHI7 will replace PCMHI6.

		PY20 Updates: PCMHI6 added as a companion metric to PACT21 which replaced PACT15. Both PCMHI6 and PACT21 are weighted at .5.<br><br>PY19 Updates: 9 revised (HIAS21, SMIE1, MPT1, Psy32, Psy34, Psy36, Psy38, SUD16, SUD4), and 1 new metric (Fam2)
	Oracle Health Integrated	FY-2021 Qtr 4
Reporting	Measure Reported on / Target	Integrated Clinical Communities with Target of \`No Target\`<br>Quality Indicator with Target of \`No Target\`<br>Strategic Analytics for Improvement and Learning with Target of \`No Target\`<br>Data due for Qtr1 on Feb 28 2025<br>Data due for Qtr2 on Apr 22 2025<br>Data due for Qtr3 on Aug 21 2025<br>Data due for Qtr4 on Nov 27 2025
	Composite Reporting	Composite Component: % depression-dxed Veterans w/ psychotherapy visit for depression, weighted (psy32) from 2016-Present<br>Composite Component: % MH-dxed Vets who had an E& M visit (pmed1) from 2016-Present<br>Composite Component: % MH-service-connected Vets in the facility catchment w/ MH care (pop6) from 2016-Present<br>Composite Component: % MH-treated patients w/ family psychotherapy visit, weighted (fam2) from 2019-Present<br>Composite Component: % of Veterans with opioid use disorder dx who received MOUD (sud16) from 2016-Present<br>Composite Component: % Pts w/ schizophrenia, bipolar disorder, or other psychoses using supported employment services (smie1) from 2016-Present<br>Composite Component: % PTSD-dxed Vets w/ psychotherapy visit for PTSD, weighted (psy38) from 2016-Present<br>Composite Component: % SMI-dxed Vets w/ psychosocial tx for SMI, weighted (psy34) from 2016-Present<br>Composite Component: % SUD-dxed Vets w/ psychosocial tx for SUD, weighted (psy36) from 2016-Present

		Composite Component: % SUD-dxed Vets who used intensive SUD treatment (sud4) from 2016-Present<br>Composite Component: % Vets w/ ICMHR-targeted dx receiving ICMHR (formerly MHICM) services (hias21) from 2016-Present<br>Composite Component: % Vets w/ PRRC-targeted dx served by PRRC (hias72) from 2016-Present<br>Composite Component: Percent of All Primary Care Patients engaged in PC-MHI (pact21) from 2020-Present<br>Composite Component: Population Access Composite for High-Level Planning (popax1) from 2022-Present<br>Composite Component: Primary Care-Mental Health Same Day Access for Initial Care (pcmhi7) from 2021-Present<br>Part of Composite: Mental Health Domain Quality (MH Balanced Scorecard) (mhq3) from 2018-Present
		Composite Component: % depression-dxed Veterans w/ psychotherapy visit for depression, weighted (psy32) from 2016-Present<br>Composite Component: % MH-dxed Vets who had an E& M visit (pmed1) from 2016-Present<br>Composite Component: % MH-service-connected Vets in the facility catchment w/ MH care (pop6) from 2016-Present<br>Composite Component: % MH-treated patients w/ family psychotherapy visit, weighted (fam2) from 2019-Present<br>Composite Component: % of Veterans with opioid use disorder dx who received MOUD (sud16) from 2016-Present<br>Composite Component: % Pts w/ schizophrenia, bipolar disorder, or other psychoses using supported employment services (smie1) from 2016-Present<br>Composite Component: % PTSD-dxed Vets w/ psychotherapy visit for PTSD, weighted (psy38) from 2016-Present<br>Composite Component: % SMI-dxed Vets w/ psychosocial tx for SMI, weighted (psy34) from 2016-Present<br>Composite Component: % SUD-dxed Vets w/ psychosocial tx for SUD, weighted (psy36) from 2016-Present

		Composite Component: % SUD-dxed Vets who used intensive SUD treatment (sud4) from 2016-Present<br>Composite Component: % Vets w/ ICMHR-targeted dx receiving ICMHR (formerly MHICM) services (hias21) from 2016-Present<br>Composite Component: % Vets w/ PRRC-targeted dx served by PRRC (hias72) from 2016-Present<br>Composite Component: Percent of All Primary Care Patients engaged in PC-MHI (pact21) from 2020-Present<br>Composite Component: Population Access Composite for High-Level Planning (popax1) from 2022-Present<br>Composite Component: Primary Care-Mental Health Same Day Access for Initial Care (pcmhi7) from 2021-Present<br>Part of Composite: Mental Health Domain Quality (MH Balanced Scorecard) (mhq3) from 2018-Present
Tech Manual	Cohort/Eligible Population	The composite measure includes measures focusing on various subgroups of mental health patients.
	Cohort/Eligible Population Inclusions	
	Cohort/Eligible Population Exclusion	
	Denominator	14 (total of weights for 16 constituent measures)
	Denominator Inclusion	
	Denominator Exclusion	
	Numerator	Total of standardized scores for constituent measures
	Numerator Inclusions	
	Numerator Exclusion	
	Denominator Exception	
	Care Modality	Not Applicable
	Rationale	
	Web Reference	MH SAIL Snapshot page

eTable 2

Outpatient MH staff to treated patient ratio definition

tablename	Attribute	Information
Definition	Measure Name	Outpatient Mental Health Staff to Treated Patient Ratio
	Measure Mnemonic	mhsa2
	Measure Coordinator	Ann S. Combs
	Measure Long Description	Onboard outpatient mental health staff per 1000 uniques receiving specialty mental health treatment
	Date of Activation	Mar 3 2020
	Date of Deactivation	

	Data Source Definition	Corporate Data Warehouse, PAID, and DSS labor mapping files
	Data Source Report	PEC Portal Tools
	Measure Notes	Based on Mental Health Onboard Clinical (MHOC) outpatient mental health FTE as calculated via collaboration between Office of Mental Health Operations and Workforce Management and Consulting. Onboard means that the provider was VA paid for hours worked in the last pay period. Outpatient refers to time spent conducting outpatient appointments. Mental Health refers to time spent providing clinical care in mental health specialty clinics (based on stop code or provider type). Clinical means time mapped to clinical in labor mapping files. FTE refers to 80 hours effort per pay period. (https://vaww.portal2.va.gov/sites/PERC/PEC_Portal/_layouts/15/xlviewer.aspx?id=/sites/PERC/PEC_Portal/SiteAssets/MHOC%20Data%20dictionary%20and%20crosswalk.xlsx)
Details	Measure - Indicator Type	Quality Indicator with Goal
	Target Definition	
	Scoring and Report Display	Quarterly
	Preferred Score Direction	Higher is Better
	Score Calculation Definition	Calculated as (a) total mental health FTE in a pay period, (b) divided by total mental health outpatients in prior quarter, (c) multiplied by 1000. (d) Average of pay period ratios is calculated to obtain score for a given quarter.
	FY Specific Annotations	Data lags 8 weeks
	Oracle Health Integrated	No
Reporting	Measure Reported on / Target	Integrated Clinical Communities with Target of `No Target`<br>Quality Indicator with Target of `7.72`<br>Data due for Qtr1 on Jan 21 2025<br>Data due for Qtr2 on Apr 22 2025<br>Data due for Qtr3 on Jul 22 2025<br>Data due for Qtr4 on Oct 21 2025

Tech Manual	Cohort/Eligible Population	All staff and patients that generate or receive any specialty mental health encounter workload.
	Cohort/Eligible Population Inclusions	
	Cohort/Eligible Population Exclusion	
	Denominator	Total facility patients treated in mental health clinics and bedsections in the four quarters prior to the one in which the numerator is obtained.
	Denominator Inclusion	
	Denominator Exclusion	
	Numerator	MH outpatient full-time equivalent (FTE; summed within facility) per a given pay period.
	Numerator Inclusions	All staff who meet the following criteria in a pay period (regardless of provider type or service line) are included in the numerator: (1) gt 0 MH outpatient encounters, (2) gt 0% mapped to direct clinical care, (3) gt 0 hours worked, and (4) not a trainee, resident, chaplain, PCP or contract employee. MH outpatient encounters exclude inpatient, homeless, radiology, laboratory, and C&P stop codes. MH outpatient encounters include all workload from psychiatrists and psychologists (per personclass), plus workload from MH outpatient stopcodes. We calculate FTE for an individual staff member by multiplying (a) proportion of patient encounters occurring in outpatient MH clinics, by (b) percentage of time allocated to direct clinical care, by (c) hours worked. We divide this factor by 80 (possible hours in a pay period) to obtain outpatient mental health FTE per staff member, which is then summed across facility to obtain the numerator.
	Numerator Exclusion	
	Denominator Exception	
	Care Modality	Not Applicable
	Rationale	Performance on measures of mental health access, quality and experience of care is generally poorer in facilities with staffing ratios below 7.72 onboard mental health clinical FTE/1000 mental health treated patients.
	Web Reference	MH SAIL Snapshot page

eTable 3

Outpatient MH population staffing ratio definition

tablename	Attribute	Information
Definition	Measure Name	Outpatient Mental Health Population Staffing Ratio
	Measure Mnemonic	mhsa3
	Measure Coordinator	Ann S. Combs
	Measure Long Description	Onboard outpatient mental health staff per 1000 facility uniques
	Date of Activation	Mar 3 2020
	Date of Deactivation	
	Data Source Definition	Corporate Data Warehouse, PAID, and DSS labor mapping files
	Data Source Report	PEC Portal Tools

	Measure Notes	Based on Mental Health Onboard Clinical (MHOC) outpatient mental health FTE as calculated via collaboration between Office of Mental Health Operations and Workforce Management and Consulting. Onboard means that the provider was VA paid for hours worked in the last pay period. Outpatient refers to time spent conducting outpatient appointments. Mental Health refers to time spent providing clinical care in mental health specialty clinics (based on stop code or provider type). Clinical means time mapped to clinical in labor mapping files. FTE refers to 80 hours effort per pay period. (https://vaww.portal2.va.gov/sites/PERC/PEC_Portal/_layouts/15/xlviewer.aspx?id=/sites/PERC/PEC_Portal/SiteAssets/MHOC%20Data%20dictionary%20and%20crosswalk.xlsx)
Details	Measure - Indicator Type	Quality Indicator without Goal
	Target Definition	
	Scoring and Report Display	Quarterly
	Preferred Score Direction	Higher is Better
	Score Calculation Definition	Calculated as (a) total mental health FTE in a pay period, (b) divided by total facility uniques in prior quarter, (c) multiplied by 1000. (d) Average of pay period ratios is calculated to obtain score for a given quarter.
	FY Specific Annotations	Data lags 8 weeks
	Oracle Health Integrated	No
Reporting	Measure Reported on / Target	Integrated Clinical Communities with Target of `No Target`<br>Quality Indicator with Target of `No Target`<br>Data due for Qtr1 on Jan 21 2025<br>Data due for Qtr2 on Apr 22 2025<br>Data due for Qtr3 on Jul 22 2025<br>Data due for Qtr4 on Oct 21 2025
Tech Manual	Cohort/Eligible Population	All staff that generate or receive any specialty mental health encounter workload. All patients that generate or receive any stay or encounter in prior 4 quarters.
	Cohort/Eligible Population Inclusions	
	Cohort/Eligible Population Exclusion	

	Denominator	Total facility patients with inpatient stay or encounter in the four quarters prior to the one in which the numerator is obtained.
	Denominator Inclusion	
	Denominator Exclusion	
	Numerator	MH outpatient full-time equivalent (FTE; summed within facility) per a given pay period.
	Numerator Inclusions	All staff who meet the following criteria in a pay period (regardless of provider type or service line) are included in the numerator: (1) gt 0 MH outpatient encounters, (2) gt 0% mapped to direct clinical care, (3) gt 0 hours worked, and (4) not a trainee, resident, chaplain, PCP or contract employee. MH outpatient encounters exclude inpatient, homeless, radiology, laboratory, and C&P stop codes. MH outpatient encounters include all workload from psychiatrists and psychologists (per personclass), plus workload from MH outpatient stopcodes. We calculate FTE for an individual staff member by multiplying (a) proportion of patient encounters occurring in outpatient MH clinics, by (b) percentage of time allocated to direct clinical care, by (c) hours worked. We divide this factor by 80 (possible hours in a pay period) to obtain outpatient mental health FTE per staff member, which is then summed across facility to obtain the numerator.
	Numerator Exclusion	
	Denominator Exception	
	Care Modality	Not Applicable
	Rationale	Performance on measures of mental health access, quality and experience of care is generally poorer in facilities with staffing ratios below 7.72 onboard mental health clinical FTE/1000 mental health treated patients.
	Web Reference	MH SAIL Snapshot page
