# Supplementary material for: Veterans Affairs Clinical Resource Hubs and Rates of Mental Health Community Care Referrals
Source: JAMA Netw Open. 2026 Feb 27;9(2):e2560084. doi: 10.1001/jamanetworkopen.2025.60084 (PMC12949438; doi:10.1001/jamanetworkopen.2025.60084)
Supplement: Supplement 2. — Data Sharing Statement [file jamanetwopen-e2560084-s002.pdf]

Data Sharing Statement

Connolly. Veterans Affairs Clinical Resource Hubs and Rates of Mental Health Community Care Referrals. *JAMA Netw Open*. Published February 27, 2026.
doi:10.1001/jamanetworkopen.2025.60084

Data
Data available: No

Additional Information
Explanation for why data not available: A deidentified dataset can be made available upon reasonable request.
